# Survival of a Maxillary Incisor in an Adolescent Male 16 Years after Its Delayed Replantation

**DOI:** 10.3390/dj7040101

**Published:** 2019-10-16

**Authors:** Roberto Biagi, Valerio Maccagnola

**Affiliations:** 1 Department of Biomedical, Surgical and Dental Sciences, School of Dentistry, University of Milan, Fondazione IRCCS Cà Granda Ospedale Maggiore Policlinico, UOC di Chirurgia Maxillo-Facciale e Odontostomatologia, 20122 Milan, Italy; 2 Private Practice of Orthodontics, Alfianello, 25020 Brescia, Italy; maccagnolacomunicazioni@gmail.com

**Keywords:** ankylosis, avulsion, infraocclusion, permanent incisor, pulpal necrosis, replantation, root resorption

## Abstract

Introduction: Recreational and sport activities, traffic accidents and human behaviour represent the main causes of trauma in young people. Case presentation: This report describes a case of a 15.2-year-old male who suffered uncomplicated crown fracture and avulsion of tooth 11 and uncomplicated crown fracture of tooth 21 due to a bicycle accident. Tooth 11 was dry stored and it was replanted 18 h after the trauma. The root was planed to remove the necrotic periodontal tissue, the pulp was extirpated before replantation and a flexible splint was applied to tooth 13 to tooth 23 for 3 weeks. A replacement root resorption of replanted tooth was suspected at the 3-month radiographic control and suffered a dramatic increase later; minimal infraocclusion, about 1 mm, was observed due to its ankylosis. Sixteen years after the trauma the patient was scheduled for an orthodontic and implanto-prosthetic rehabilitation. Conclusion: Delayed replantation usually has a long-term poor prognosis, so it is very important to promote awareness regarding emergency management modalities in dental traumatology especially among parents, school teachers, and coaches that are usually present at the site of the accident.

## 1. Introduction

Traumatic avulsion of teeth is a very serious assault on the gingiva, the periodontal ligament and the pulp. It occurs with a range from 0.5 to 3% of injuries in permanent teeth, most often in children and adolescents, because a relatively resilient alveolar bone provides only minimal resistance to extrusive forces [1].

Recreational and sport activities, traffic accidents and human behavior, such as risk-taking children, children being bullied, emotionally stressful conditions, obesity, and attention-deficit hyperactivity disorder, represent the main causes of trauma in young people. Oral factors, particularly increased overjet with protrusion and inadequate lip coverage, amplify the risk of traumatic dental injuries [2].

Pulp and periodontal healing depends on the stage of tooth development, time, and type of extra-alveolar storage, therefore, a tooth with an open apex has a greater potential for pulpal revascularization and tissue regeneration because it may contain a rich blood supply and stem cells [3,4]; and pH and osmolarity of the storage medium should be compatible with the survival of periodontal ligament cells [5,6]. Moreover, the knowledge of emergency care at the scene of dental trauma is resolutory of treatment outcome [1,7].

Recall visits allow for an early diagnosis and treatment of complications like pulp necrosis or root resorption that may occur long after the trauma. 

A case of a 15.2-year-old male who suffered a facial trauma following a bicycle accident is reported with a 16-year follow-up.

## 2. Case Presentation

**Figure 1:**
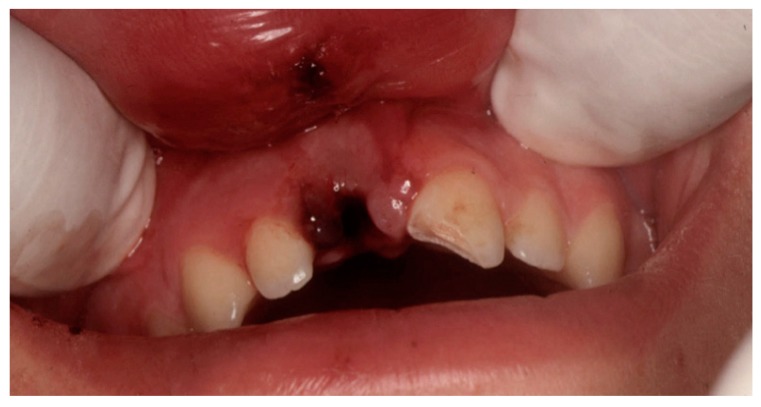
Intraoral view at the time of traumatic injury.

A healthy 15.2-year-old male arrived at the dental office coming from the Hospital Emergency Unit 18 h after a facial trauma due to a bicycle accident. Intraoral examination revealed avulsion of tooth 11 and an uncomplicated crown fracture of tooth 21 (Figure 1).

**Figure 2:**
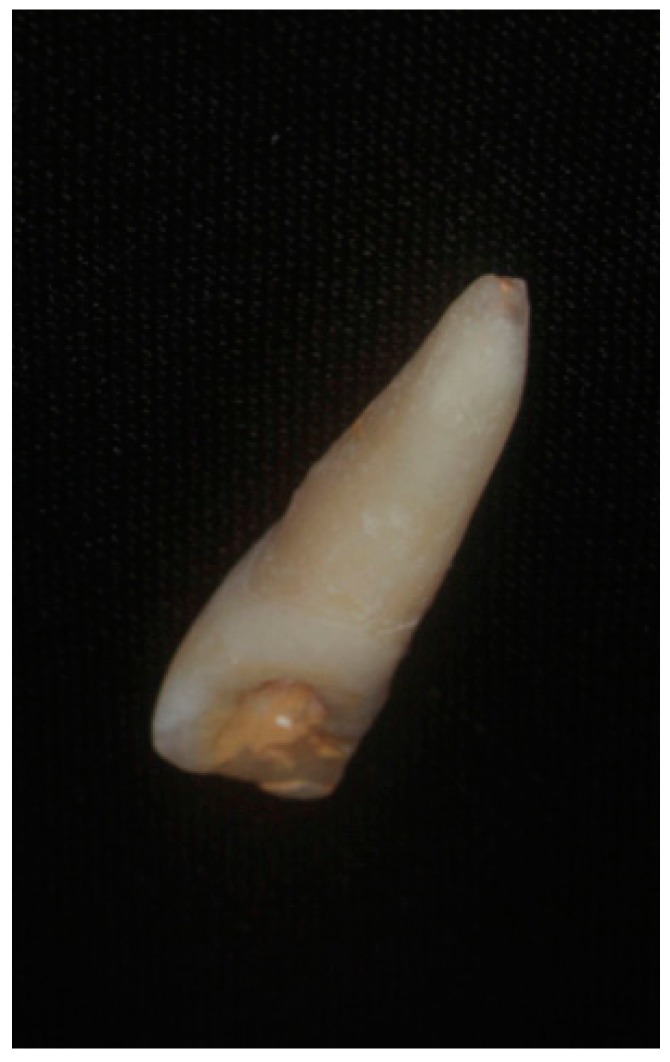
Tooth 11 after root planing, pulpectomy, and filling with gutta-percha and sealer.

The fragment of tooth 21 was discovered inside the swollen and lacerated upper lip. No other oral injury was detected clinically. Examination of the avulsed tooth showed an uncomplicated crown fracture and necrotic periodontal ligament cells due to the prolonged dry storage. After the parents’ informed consent, the root of the avulsed tooth was planed to remove the necrotic periodontal tissue, and was then filled with gutta-percha and sealer after pulpectomy (Figure 2).

**Figure 3:**
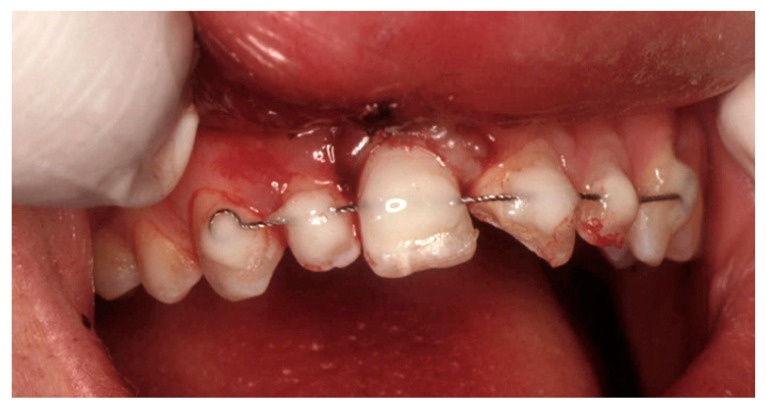
Intraoral view after replantation and functional splinting of tooth 11.

The fractured crown was restored with composite resin. A local anaesthetic was administered to remove the contaminated coagulum from the socket prior to pushing back the tooth with a gentle pressure. A functional splinting with an orthodontic 0.014-inch braided stainless steel wire and composite resin was positioned from tooth 13 to tooth 23 (Figure 3) and left in place for 3 weeks. Fragment of tooth 21 was not utilized to restore its fractured crown, and a seal against bacterial invasion into its dentinal tubules was created using a dentin bonding agent. The proper positioning of tooth 11 was confirmed with a periapical radiograph. Tetanus prophylaxis was not necessary. A 7-day treatment of systemic amoxicillin (2 g for day) and an analgesic on demand were prescribed at the Hospital Emergency Unit. Finally, strict instructions were given to the patient about soft diet for 1 week and oral hygiene (soft toothbrush after each meal and mouth rinsing twice a day with 0.12 chlorexidine) for the entire splinting period.

**Figure 4:**
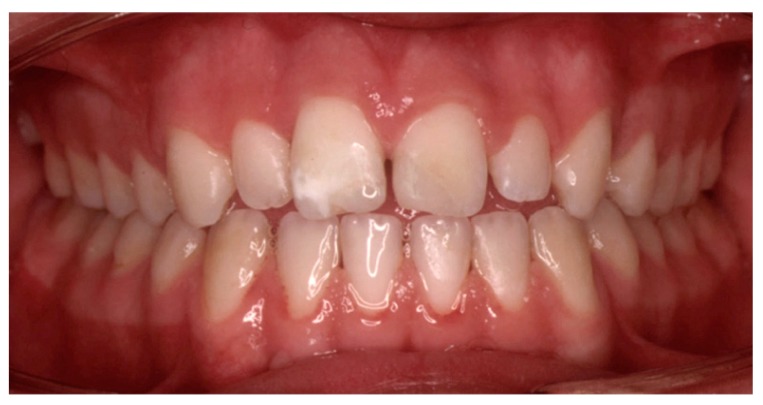
Intraoral view when the splint was removed (three weeks after injury).

The splint was removed 3 weeks later and the fractured crown of tooth 21 was restored with composite resin (Figure 4).

**Figure 5:**
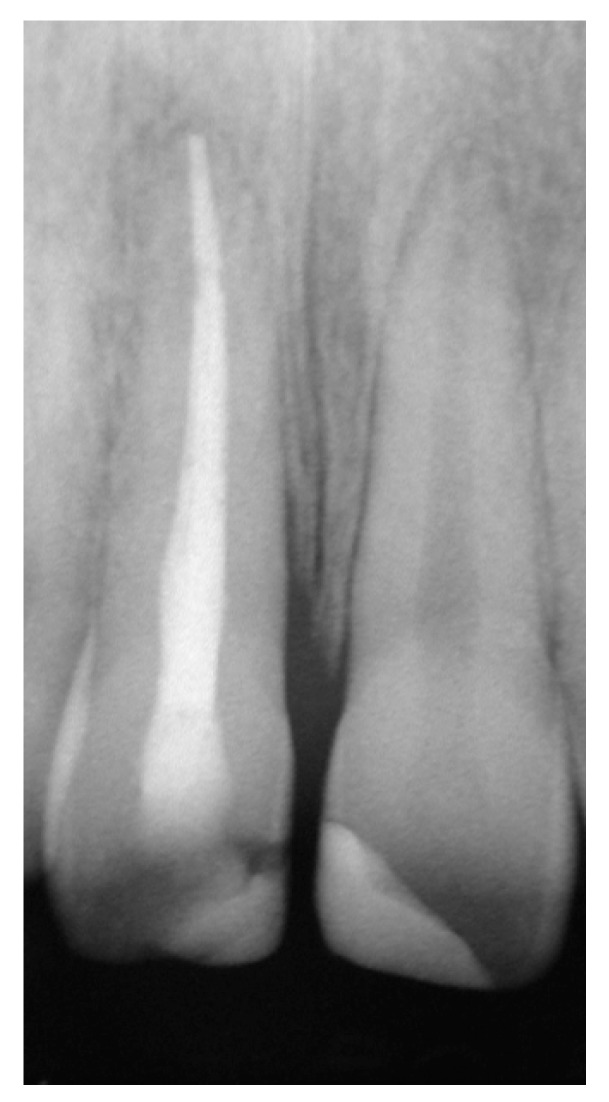
Rx at the 3-month recall visit.

**Figure 6:**
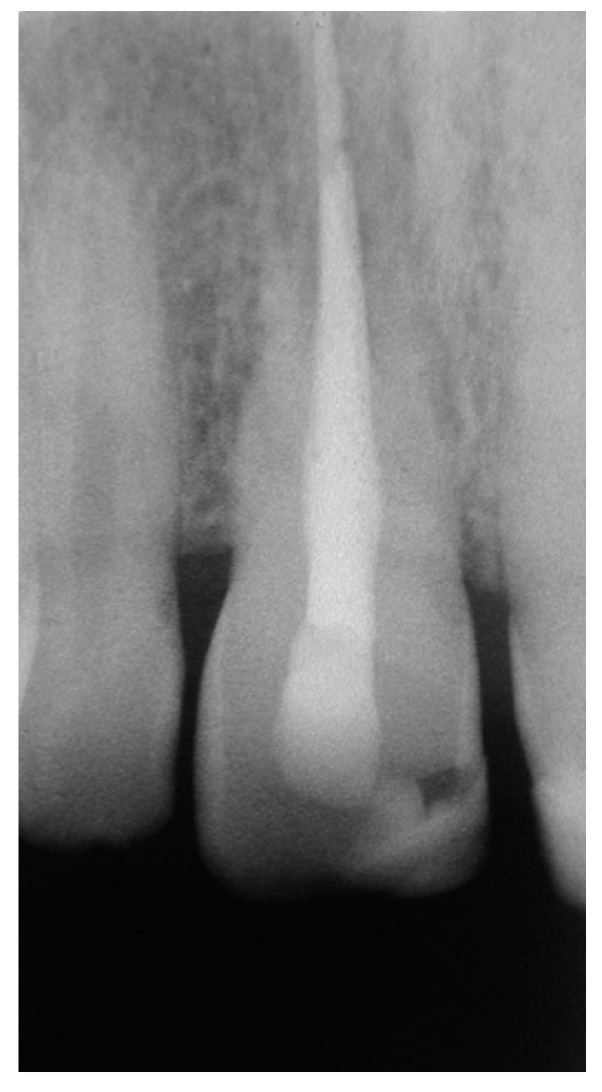
Rx at the 2-year recall visit.

**Figure 7:**
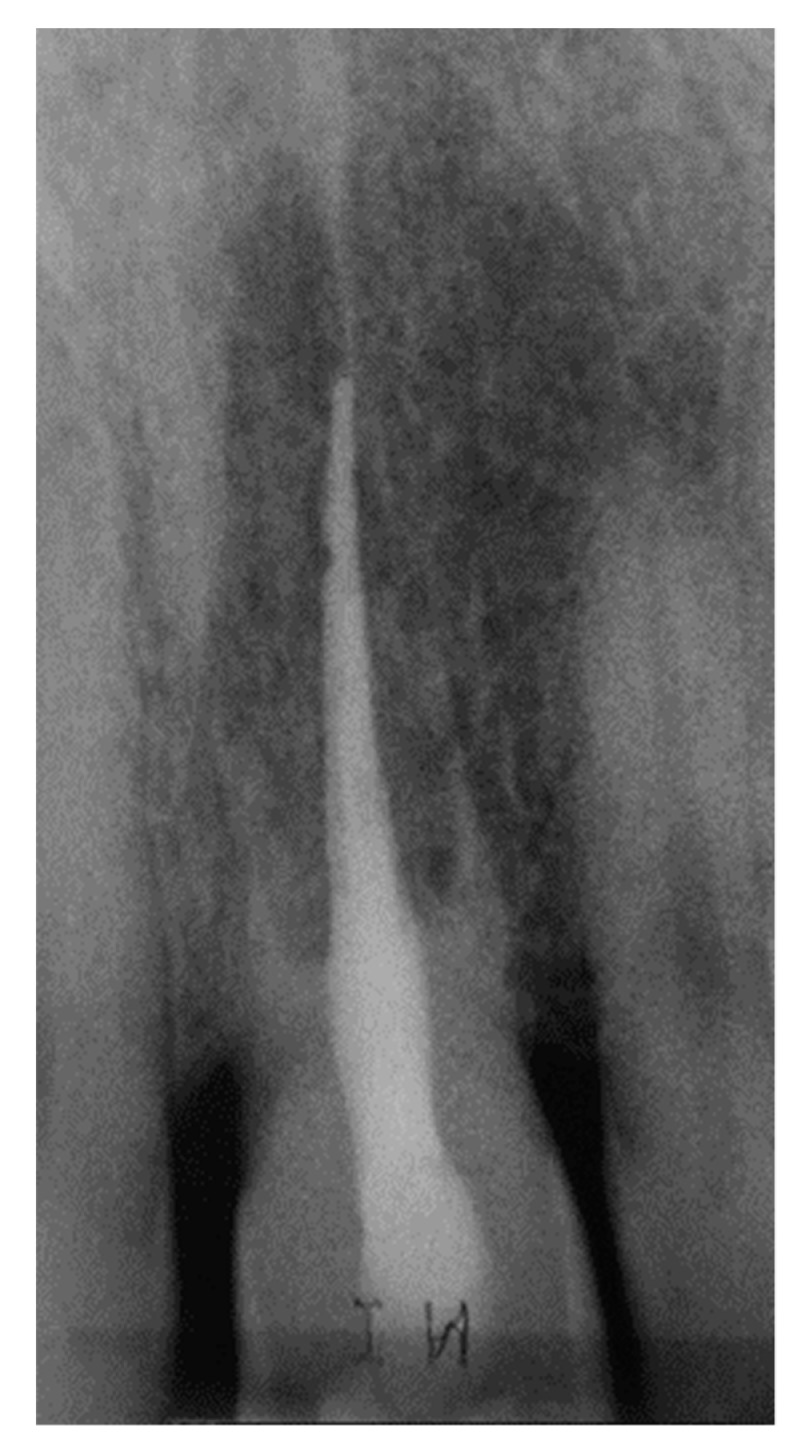
Rx at the 4-year recall visit.

**Figure 8:**
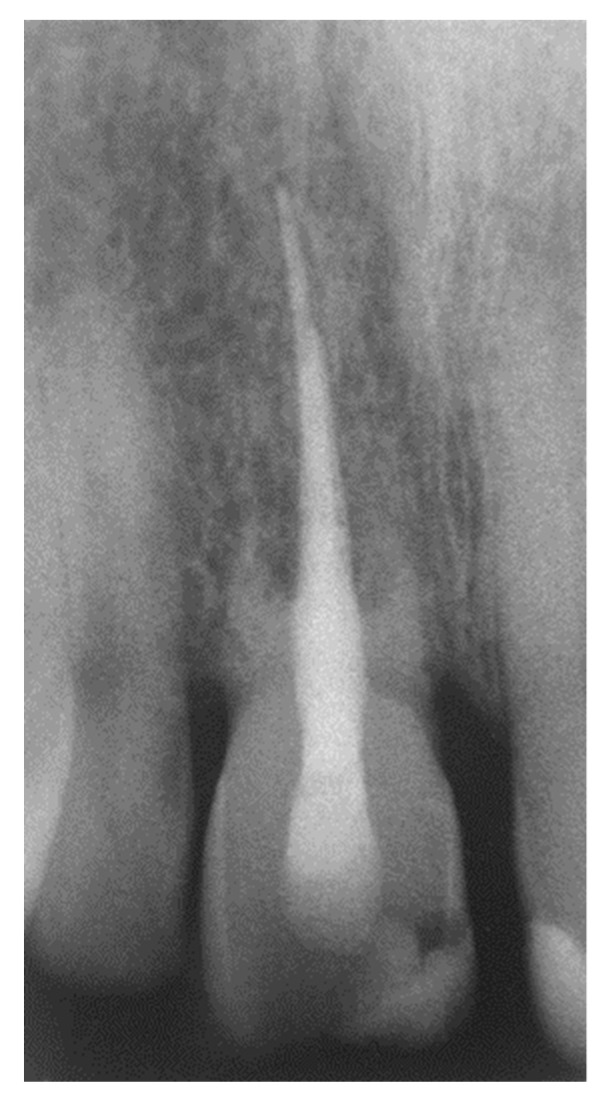
Rx at the 7-year recall visit.

**Figure 9:**
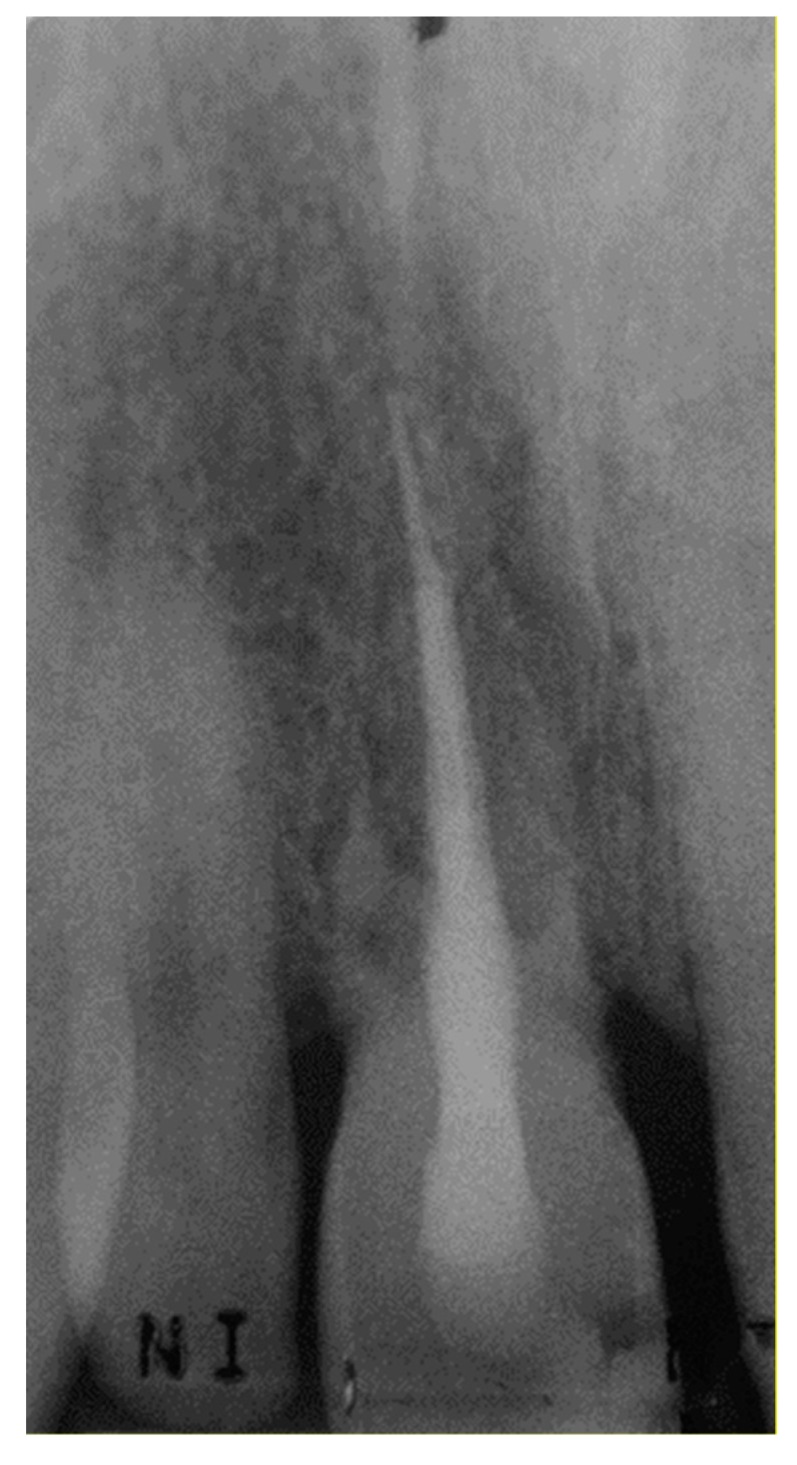
Rx at the 10-year recall visit.

The importance of recall visits was emphasized to the patient and the parents, so the patient was scheduled for a follow-up according to the International Association of Dental Traumatology (IADT). Due to the prolonged extra-alveolar dry storage (about 18 h) and the age of the patient, ankylosis and infraocclusion of tooth 11 were expected. A replacement root resorption of the replanted tooth was suspected at the 3-month radiographic control (Figure 5), and dramatically increased later (Figure 6, Figure 7, Figure 8 and Figure 9); infraocclusion about 1 mm was observed. Due to ankylosis and discoloration of the crown, and an anterior open bite, the patient was encouraged to undergo a comprehensive treatment that he previously always avoided. 

**Figure 10:**
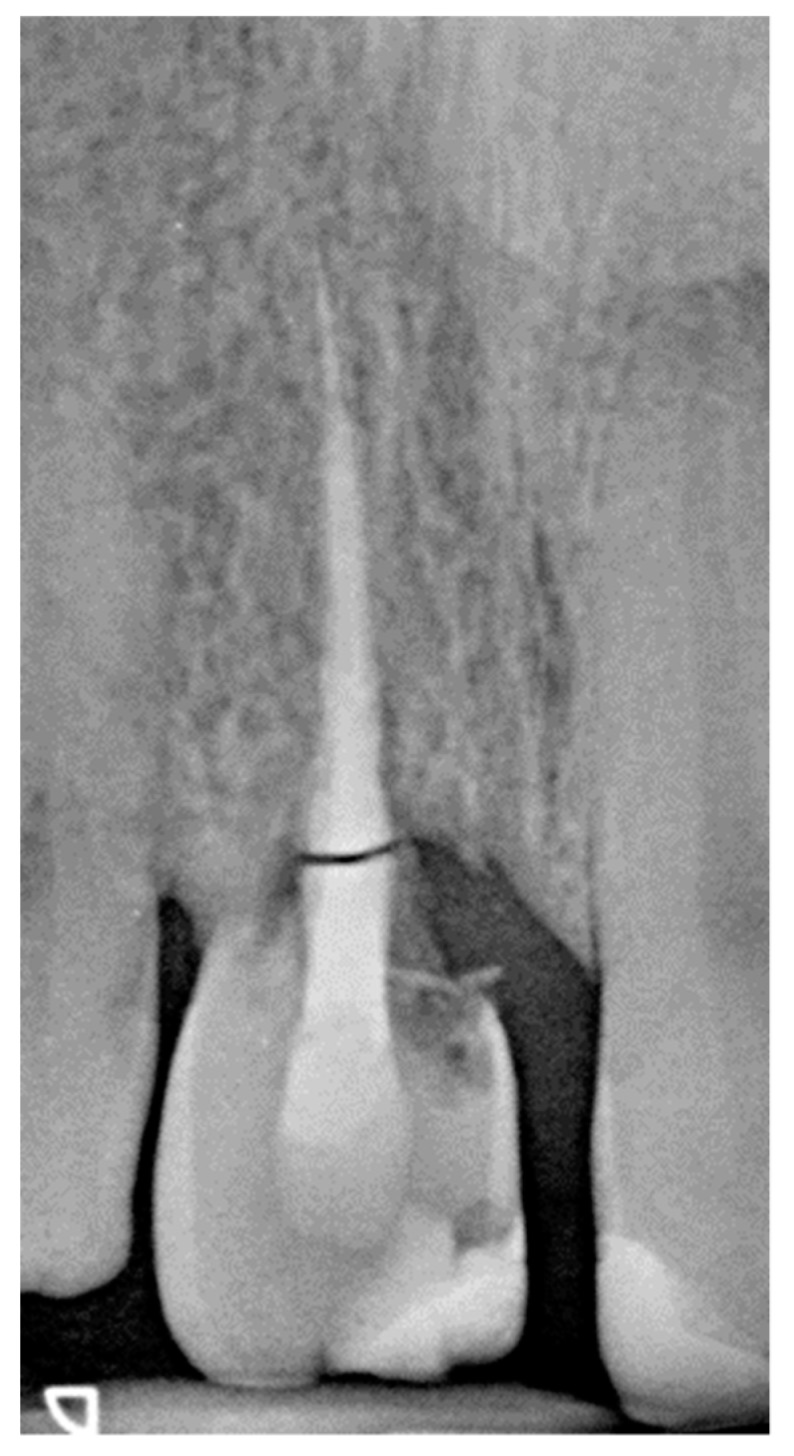
Rx at the 16-year recall visit.

**Figure 11:**
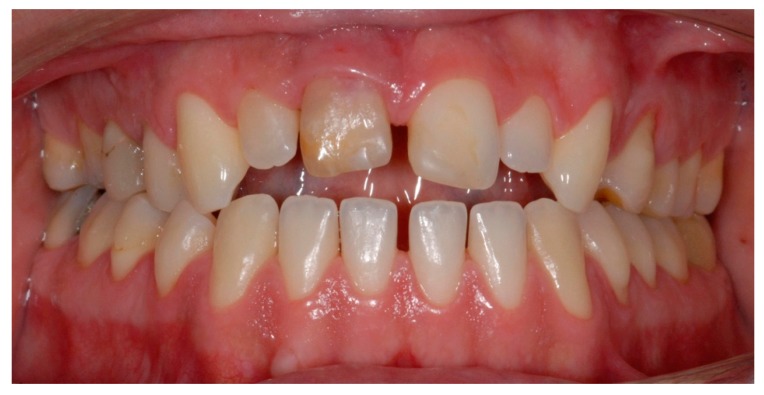
Intraoral view 16 years after delayed replantation of tooth 11.

Finally, 16 years after the trauma (Figure 10 and Figure 11) the patient was scheduled for an orthodontic and implanto-prosthetic rehabilitation.

## 3. Discussion

The prognosis of avulsed teeth is inevitably affected by appropriate first aid at the site of injury. Immediate replantation is the best practice for avulsed permanent teeth. If it is not feasible, the teeth must be stored in the mouth of the patient inside the lip or cheek or in a container with saliva for a short period of time, no longer than 30 min. Saliva is a hypotonic solution; its osmolality (60–70 mOsm/kg) is much lower than the physiologic one, causing cell swelling, which stretches the cell membrane, and potentiates the effect of bacterial products and toxins present in itself. As saliva is always available, it should be considered as a temporary medium to avoid dry storage. A combination of brief storage in saliva is recommended, with subsequent storage in a physiologic medium, rather than storage in saliva only. The ideal biological medium is Hank’s Balanced Salt Solution (HBBS), but its availability near the site of an accident is doubtful. HBBS has a pH balanced at 7.2, an osmolarity of 320 mOsm/kg, and preserves viability, mitogenicity and clonogenic capacities of periodontal ligament cells for up to 24 h. Based not only on periodontal ligament cell viability, but also on practical considerations such as low cost and high availability, milk is one of the most commonly used and recommended storage media. It has a pH of 6.5–7.2 and an osmolarity of 270 mOsm/kg. Being a gland secretion, milk contains epithelial growth factor, which stimulates the proliferation and regeneration of epithelial cell rests of Malassez, and activates the alveolar bone resorption. Pasteurized and refrigerated fresh milk with lower fat is preferable. It can effectively store the tooth for up to 6 h, although 3 h is considered the threshold storage time, and it is as effective as HBBS for up to 1 h. [3,5,6]. Unfortunately, people present at the site of an accident rarely know how to manage an avulsed tooth, so usually the replantation is followed by complications, inflammatory and replacement resorption due to dry storage. In this case, the patient was brought to a Hospital Emergency Unit, but he did not receive any dental treatment and was referred to a private office of a dental practitioner, so only 18 h after the trauma the tooth was replanted. As dental practitioners are rarely present in emergency units, it is inevitable that medical physicians will sometimes provide dental treatment, but if emergency medical physicians have not received prior training in the management of traumatic teeth avulsion injuries, they are unable to provide appropriate emergency treatment for such an injury [8]. Moreover, a recent study shows a high level of awareness among medical practitioners about infection and inflammation, and a low level of awareness about preserving tooth vitality [9]. Guidelines of IADT should assist in decision making in a Hospital Emergency Unit, and their application could maximize the chances of a favourable outcome [10]. The tooth was replanted after a prolonged dry extra-alveolar period with a poor prognosis. The optimal time for pulpal extirpation after replantation has not been established in human clinical studies, but the current clinical guidelines support a period within 10–14 days after replantation to prevent an early infection-related resorption, and therefore the risk of an early tooth loss [11,12]. In this case, because the dry time was more than 60 min, the immediate definitive endodontic treatment was performed. The damage to the periodontal ligament cells will elicit a severe inflammatory response over a diffuse area on the root surface and through physiologic bone re-contouring, the entire root will be replaced by bone, a process which has been termed replacement resorption [3]. A complete resorption of the root takes 3–7 years in patients aged 8–16 years, and longer in older patients because the rate of resorption is much more rapid in children [1,13]. In this case, tooth 11 survived 16 years after its delayed replantation and only a “minimal” infraocclusion, according to Malmgren and Malmgren’s classification (Index 1: < 1/8 of the crown height, compared to the homologous maxillary incisor with healthy periodontal ligaments), due to its ankylosis was observed [14]. Ankylosis remains the predominant clinical problem that affects replantation success, particularly in growing patients. In children and adolescents, ankylosis of permanent incisors creates a localized arrest in the growth of the alveolus and the infraocclusion is greater before the pubertal growth spurt for the higher metabolic rate [1,14,15]. In this case, replantation was performed at 15.2 years of age, after the pubertal growth spurt, so a minimal infraocclusion was expected with low esthetic and functional risks and a request of a simple restorative care. Finally, a shorter flexible splinting time is preferred up to 2 weeks, according to the guidelines of the IADT [10], but a longer period seems not to affect the likelihood of successful periodontal healing after replantation [16]. 

## 4. Conclusions

Delayed replantation usually has a long-term poor prognosis. The follow-up of a replanted tooth observed in the present case has rarely been described in literature. The possibility of a very long survival encourages dentists to attempt this procedure to fulfill the esthetic, functional, and psychological needs of the patients who are in late adolescence or adulthood when they sustain their injury. Conversely, immediate replantation at the site of the accident or correct maintenance of an avulsed tooth in a physiologic storage medium are resolutory for treatment outcome. Therefore, it is very important to promote awareness regarding emergency management modalities in these severe and unexpected injuries, not only among emergency healthcare professionals, but especially among parents, school teachers, and coaches that are usually present at the site of the accident, through direct education, via press or by mass media campaigns.
